# Navigating Lone Parenthood Over Time: A Qualitative and Vulnerability Life-Course Approach

**DOI:** 10.1177/0192513X251393152

**Published:** 2025-11-03

**Authors:** Benjamin Moles, Laura Bernardi

**Affiliations:** 1 LIVES Swiss Centre of Expertise in Life Course Research, 27213 University of Lausanne, Lausanne, Switzerland

**Keywords:** family sociology, lone parenthood, vulnerability, life course, qualitative research

## Abstract

Despite its increasing diversity, lone parenthood is still largely treated as a homogeneous group, with research often assuming a causal link between this family structure and sociodemographic disadvantages and paying little attention to how lone parents navigate and respond to these challenges over time. Drawing on qualitative longitudinal data from the Swiss panel study *Multiple Paths of Lone Parenthood* (2012–2022), this article proposes a dynamic and multidimensional model of lone parenthood, grounded in a vulnerability life-course perspective. It examines the lived experiences and developmental trajectories of 20 lone mothers across 100 in-depth biographical interviews. The analysis identifies three distinct phases in the lone parenthood experience and outlines four ideal-type trajectories, highlighting how mothers navigated life domains, such as employment, housing, health, and caregiving, over time. Although most lone mothers initially faced compounded stressors across multiple domains, many demonstrated recovery and adaptation—except in cases of chronic vulnerability. The findings indicate that resilient and non-vulnerable mothers often achieve and maintain stability, whereas those with vulnerable trajectories experienced renewed stressors over time. This study challenges static understandings of lone parenthood by emphasizing its temporal, relational, and situational complexity. It calls for policy interventions that combine early and sustained support, recognizing the heterogeneity of lone parents’ pathways and the shifting nature of their vulnerabilities over time.

Lone parenthood has become an increasingly common family form in the so-called advanced societies in recent decades; nevertheless, from a biographical perspective, it remains unexpected and unintended and is one of the most critical life-course transitions for parents and children. Nowadays, union instability and marital disruptions are the main reasons an adult raises a child alone (Struffolino & Bernardi, 2017). In the context of the second demographic transition, the high rate of separations and divorces is leading to an increase in the diversity within the lone-parent family population. Meanwhile, transitions *out* of lone parenthood through repartnering have accelerated in recent years, making lone parenthood a *transitory* state for most (Bernardi & Larenza, 2018). The combination of the democratization of parental union break-ups and the accelerated dynamics of the transitions *into* and *out* of lone parenthood makes it a highly heterogeneous and dynamic family experience.

However, despite these transformations, most family and social policy research portrays lone parents as a homogeneous group (Zagel, 2023) and, more or less implicitly, establishes a causal relationship between this particular family structure and disadvantages associated with it. Such disadvantages, especially for lone mothers, include higher risks of unemployment, poverty, and poorer health and well-being (Bradshaw, 1996; Coltrane & Adams, 2003; Demo & Fine, 2010; Glass et al., 2016; Hetherington, 2014; Struffolino et al., 2020; Van Gasse & Mortelmans, 2020; Walsh, 2016; Bernardi et al., 2018). Research has also highlighted negative outcomes for children, including lower educational achievement, reduced emotional well-being, and a greater likelihood of becoming lone parents themselves (Amato, 2005; Francesconi et al., 2006; McLanahan & Sandefur, 2009). Most of these findings are based on cross-sectional quantitative research; however, longitudinal research has challenged the causal nature of the relationship between lone parenthood and negative outcomes (Baranowska-Rataj et al., 2014). By contrast, qualitative research has provided insights into the heterogeneity of lone parents’ experiences and focuses more on the agentic dimension of lone parenthood (Caragata et al., 2021; Hamilton, 2012; Larenza, 2019). Although these approaches have improved the understanding of transformations within and challenges for the lone-parent population, they have largely focused on addressing the “symptoms” (Zagel, 2014; Mortelmans & Defever, 2018). Therefore, they have often overlooked the dynamic nature of the challenges associated with lone parenthood—particularly how lone parents adapt to, cope with, and overcome vulnerability across life domains and over time.

From a life-course and qualitative perspective, this study aims to contribute to the field by examining the trajectories of 20 lone mothers over more than 10 years (2012–2022) living in Switzerland. First, we apply the key concepts of *vulnerability* and *human agency*. We show the importance of considering vulnerability as a dynamic interplay between stressors *and* resources across different life domains. Simultaneously, we emphasize the role of human agency by acknowledging lone parents’ ability to actively shape and improve their circumstances over time.

Building on this conceptual framework, we identify three distinct phases in the experience of lone parenthood and define four ideal-type trajectories, advancing a dynamic and empirically grounded model of lone parenthood. This model shows that although most lone mothers encounter significant stressors at the beginning of their trajectories, many react as individuals with agency, adapting to adverse situations, ultimately improving their overall circumstances. Nevertheless, renewed stressors lead some lone mothers to a second crisis. We interpret these different dynamics through the conceptual framework of *latent* and *manifest* vulnerability (Spini et al., 2017), showing that an insufficient “recovery” phase may lead to new and more manifest forms of vulnerabilities.

In the remainder of this article, the first section reviews the research on lone-parent families and presents the core principles and concepts from the theoretical framework of life course applied in this study. The subsequent sections briefly describe the Swiss context and introduce the data and methodological approach. The findings are then presented and discussed. The concluding section presents the limitations of this study and future avenues for research.

## Theoretical Background

To explain the disadvantages that most lone parents, and especially mothers, face, research has provided three main explanations. First, focus on individuals’ sociodemographic characteristics before the transition suggests a *social selection* mechanism in which individuals from lower socioeconomic backgrounds are more exposed to family instability and, consequently, more likely to experience lone parenthood—a condition that further amplifies their preexisting disadvantages (McLanahan, 2004, 2014). Second, causal mechanisms have been identified that link the challenges of lone parenthood to the absence of a co-parent, which constrains financial resources, caregiving capacity, time flexibility, and access to emotional and psychological support (Amato, 2000; Amato & Gilbreth, 1999; Brady & Burroway, 2012; Broussard et al., 2012; Glass et al., 2016; Hübgen, 2018; McLanahan & Sandefur, 2009; Millar & Ridge, 2009). Third, scholars have emphasized the critical role of social policy and institutional contexts in shaping the living conditions of lone parents. They have highlighted the limitations of the “welfare-to-work” policies and underscored the importance of integrating broad access to public resources and services (e.g., childcare and parental leave) with private transfers (e.g., child, family, and housing benefits) to mitigate the disadvantages faced by lone parents (Cook, 2012; Larenza, 2019; Nieuwenhuis & Maldonado, 2018).

Longitudinal research has offered a more nuanced explanation of lone parenthood and its association with negative outcomes. By linking the pre- and post-transition stages, it has provided clearer insights into the effects of the transition to lone parenthood while highlighting the diversity within the lone-parent population and demonstrating that their experiences and challenges are not uniform and can evolve (Baranowska-Rataj et al., 2014). Additionally, research adopting a life-course perspective offers a more comprehensive understanding of the complexities involved, emphasizing the *multidimensional* nature of the challenges lone parents face across life domains (Recksiedler & Bernardi, 2019; Struffolino et al., 2020).

The responses and coping strategies of families have been examined. From a family systems theory perspective, researchers have developed different models to explain family responses and coping mechanisms to face disruptive events such as separation and divorce^1^ (Emmers-Sommer et al., 2003; Greenwood, 2014; Hill, 1949, 1958; Moore, 2011). Recently, Van Gasse and Mortelmans (2020) proposed a six-stage model of post-separation household reorganization, beginning with the decision to separate and the initial transition, followed by a period of uncertainty and temporary reorganization of daily life, ultimately leading to the establishment of a new family equilibrium. Although this research highlights the agentic dimension of lone parenthood, it presents certain limitations, suggesting a deterministic progression model—from crisis to recovery—overlooking cases that do not follow such a linear progression.

Fewer studies have emphasized the role of informal support systems in helping individuals navigate the challenges associated with the transition to lone parenthood. Some highlight the particular role of individual skills and psychological dispositions in managing difficulties (Caragata et al., 2021; Cheeseman et al., 2011; Lenette et al., 2012; Taylor & Conger, 2017), while others emphasize the importance of personal networks—family, friends, or neighbors—in providing access to essential resources (Harknett, 2006; Harknett & Hartnett, 2011; Keim, 2018; McLanahan et al., 1981; Niepel, 1994). However, these approaches are scarce and do not offer insights into how these support systems evolve as lone parents undergo subsequent family and life transitions across different domains and potentially face new challenges.

Overall, the literature on lone parents lacks a comprehensive approach that integrates both stressors and agency, a multidimensional understanding of stressors across life domains, and a dynamic perspective that captures these dimensions over time. To address this gap, first, the next subsection introduces the theoretical perspective on life course as a suitable and comprehensive framework for advancing the understanding of lone mothers’ trajectories.

### Analyzing Lone Mothers’ Trajectories Through a Vulnerability Life-Course Perspective

As a theoretical paradigm, the life-course perspective provides a framework for examining the interplay between individuals, life domains, and broader social and historical contexts over time (Elder, 1998; Shanahan et al., 2016). This study draws on two of its core concepts: *human agency* and *vulnerability*. Human agency refers to the idea that individuals shape their life course through choices and actions within the opportunities and constraints imposed by history and social structures (Elder, 1994). Building on Bernardi et al. (2019), we assume that individuals, consciously or unconsciously, strive to enhance or maintain their physical and mental well-being while avoiding significant losses. From this perspective, this study theoretically assumes lone mothers as active agents who continuously work to improve both their circumstances and those of their children.

Following Spini et al. (2013), we apply the concept of vulnerability as a “weakening process and a lack of resources in one or more life domains that, in specific contexts, expose individuals or groups to (1) negative consequences related to sources of stress, (2) an inability to cope effectively with stressors, and (3) an inability to recover from stressors or to take advantage of opportunities by a given deadline” (p. 19). From this perspective, vulnerability can be understood as a situation in which an individual lacks sufficient resources to navigate a critical transition or event, such as becoming a lone parent. However, by framing vulnerability as a dynamic interplay between stressors *and* resources, this definition acknowledges the potential for individuals, including lone parents, to improve their circumstances by mobilizing available or new resources.

We differentiate between *manifest* and *latent* vulnerability. *Manifest vulnerability* refers to situations where scarce resources result in a limited ability to react to stressors, making individuals or groups immediately exposed to losses when confronted with additional demands. *Latent vulnerability*, by contrast, describes a more hidden state in which resources are already insufficient, but negative outcomes have not yet become socially or institutionally apparent. Latent vulnerability can be understood as a process of fragilization—a gradual weakening that may remain invisible for some time but increases the risk of more severe consequences if new stressors occur (Spini et al., 2013, 2017). This distinction underscores how vulnerability can exist in less visible forms before becoming manifest, which is particularly relevant for analyzing lone-parent trajectories where stressors often can accumulate until they reach a critical threshold.

This study also mobilize the *multidimensional* nature of vulnerability, that is, the notion that vulnerability is embedded within specific interdependent social contexts, such as the life domains of employment, childcare, health, and housing. Individuals may face distinct challenges across different life domains (Spini et al., 2013, 2017). Moreover, this study considers the assumption that individuals have resources (e.g., money, time, energy, human capital, relations, or the psychoemotional dimension) to invest in specific domains, which influences the decision-making process related to each domain, thereby creating spillover effects, crucial to understanding how vulnerability processes arise and evolve (Bernardi & Bolano, 2023; Diewald, 2012; Diewald et al., 2006; Hanappi et al., 2015; Schüttengruber et al., 2023).

Overall, by grounding this study in a theoretical framework of life course and concepts of human agency and vulnerability, our approach provides a comprehensive and dynamic understanding of how lone mothers’ circumstances evolve. It highlights the challenges they face and their capacity to adapt and overcome adversity across life domains. These experiences are heterogeneous: the way in which lone mothers navigate difficulties is shaped by the sociocultural and institutional settings of their lives. The following section examines the Swiss context, where lone parenthood is situated within a conservative family and gender regime.

## The Swiss Context

Switzerland, a conservative family and gender regime (Rossier et al., 2023), is a strongly normative environment where the heterosexual married couple remains the dominant family form, often marginalizing alternative configurations, including lone parenthood; childcare responsibilities continue to fall primarily on women, both within marriage and after separation (Bornatici et al., 2020; Engeli & Roca i Escoda, 2012).

The *modified male breadwinner* model in Switzerland is characterized by a combination of fiscal policy and weak policies aimed at reconciling paid work and care (e.g., a scarce parental leave system or an undersupplied and expensive childcare system). In this context, women do not have any incentive to work full-time and typically reduce their employment to part-time after the birth of their first child, while men continue to work full-time (Le Goff & Levy, 2016). This adds up to other women-specific penalties in the labor market, such as the gender pay gap and occupational segregation (Struffolino & Mortelmans, 2018).

Separation and divorce are still particularly critical life-course transitions for women in Switzerland, thus exacerbating their already gendered individual and family trajectories. Full custody is still primarily granted to mothers^2^, who therefore encounter an asymmetrical distribution of rights and responsibilities that characterizes most of these arrangements^3^. These asymmetries, together with limited mechanisms to enforce their rights when biological fathers do not comply with their responsibilities, place lone mothers in a particularly delicate situation (Moles-Kalt et al., 2024). Additionally, the particular gendered institutional framework intensifies the work–care dilemma faced by lone mothers, frequently confronted with the choice of participating in the labor market—often with minimal public childcare support—or remaining unemployed and relying on social assistance, which may provide more time for caregiving (Struffolino et al., 2020).

Therefore, the Swiss context presents unique challenges for lone mothers who do not conform to these normative standards and serves as a particularly compelling case for examining lone mothers’ trajectories across life domains.

## Data and Methods

We used data from the project *The Multiple Paths of Lone Parenthood*, a qualitative longitudinal study on the transition to lone parenthood that followed the same families over more than a decade (2012–2022) in French-speaking Switzerland. This study is based on an analysis of the prospective narrative interviews conducted over the five project waves (W1–W5). Each wave was thematically focused, but key themes were explored in all waves, including (1) subsequent family transitions (e.g., repartnering and family recomposition); (2) changes in other life domains (e.g., childcare, employment, residence, and health); and (3) the relationship with the noncustodial parent (e.g., custody, support payments, everyday organization, and relationship with the child)^4^.

For W1, the criteria for inclusion in the sample (*N* = 40) were having sole or main custody of children 10 years old or younger and not living with any other adult at the time of the interview. The participants were recruited through a multiple-entry sampling strategy combined with limited snowballing (Sulaiman-Hill & Thompson, 2011) to avoid an overrepresentation of participants from the same socioeconomic background and to increase the proportion of those with a lower socioeconomic background (Sadler et al., 2010). We also limited the interviews to parents who had experienced a relatively recent transition to lone parenthood (i.e., within 1–5 years).

Over time, many families passed through several transitions, transforming the initial sample of lone parents into more heterogeneous family configurations^5^. In Wave 1, the average age of children was 5.75 years. Despite our efforts in recruitment, participants of lower socioeconomic backgrounds were underrepresented in the sample from the beginning and selectively dropped out in the subsequent waves.^6^ Due to attrition, 23 lone parents remained in the sample in Wave 5.

We focused on lone motherhood occurring through separation or divorce and therefore excluded the two fathers and the widow from the sample; thus, the final sample included 20 lone mothers (W1–W5). Except for Wave 4—during which interviews were conducted remotely via telephone or videoconferencing tools due to the COVID-19 pandemic restrictions—all interviews were conducted face-to-face, primarily in participants’ homes. All interviews were audio-recorded, with those in Wave 4 being either audio- or video-recorded. On average, the interviews lasted 90 min but were slightly shorter in Wave 4. All interviews (W1–W5) were transcribed and anonymized. Hence, the participant names are pseudonyms. The excerpts presented in the findings were translated from French using artificial intelligence tools and subsequently revised by the authors.

### Analytical Strategy and Methods

The analytical strategy involved both cross-sectional and longitudinal approaches, applied within each case and across cases (Hollstein, 2021; Lewis, 2007; Neale, 2019, 2021; Thomson, 2007; Vogl et al., 2018)^7^. The initial coding process of each case followed the principles of reflexive thematic analysis (Braun & Clarke, 2006, 2019) and had a basic predefined coding system with two levels of codes for each life domain (employment, childcare, housing, health, partnership, and ex-partner): (1) main event or transition; and (2) situation before transition. The five waves of data analysis had this same structure. The coding system was open and flexible and was conducted using the NVivo software. Subsequent rounds of coding were conducted by the article’s first author, followed by discussions in team meetings during a joint analysis of selected cases. Revisions were then incorporated into NVivo.

To overcome challenges associated with conducting longitudinal qualitative research and the limitations of using thematic analysis—namely, its tendency to fragment narratives into discrete themes and thereby overlook not only the temporal processes and dynamics that shape trajectories but also the specificities of individual trajectories that do not easily fit into shared patterns (Hollstein, 2021; Neale, 2021; Saldana, 2003; Thomson & Holland, 2003)—we developed two additional analytical tools: the case history files (Appendix C) and the graphical representation of cases (Appendix D). Both tools were essential for synthesizing information within and across cases, both cross-sectionally and longitudinally; they provided a much clearer visual understanding of each case and their comparisons. Drawing on Qualitative Comparative Analysis (QCA), which compares cases by the degree to which they share specific conditions or outcomes (Ragin, 1987), we applied a crisp set approach. Crisp sets code conditions in a binary way (0 = absence, 1 = presence). Using this logic, we developed tables that capture the stressors and resources of each participant at the beginning of their trajectories, in order to synthesize the cross-case comparison and initiate the grouping of different trajectories (Hollstein & Wagemann, 2014; Mello, 2021) (Appendix E)^8^.

## Findings

Table 1 synthesizes the model of lone parenthood emerging from the analysis. The findings reveal three distinct phases in the lone parenthood trajectory: (1) disruption and resource activation, (2) steadying the ship: navigating the adjustment phase, and (3) settling unevenly: late adjustments and unequal recovery. Building on the characteristics of these phases, we define four typologies of lone mother trajectories: non-vulnerable, resilient, vulnerable, and chronically vulnerable^9^. These categories reflect varying levels of stability, resource accumulation, and long-term adaptation, offering a comprehensive framework for understanding the diverse experiences of lone mothers over time.

**Table 1.** Main Characteristics of the Three Phases and Typology of Lone Mothers’ Trajectories^10^

Phase 1. Disruption and resource activation	Phase 2. Steadying the ship: navigating the adjustment phase	Phase 3. Settling unevenly: late adjustments and unequal recovery	Trajectory ideal types	Participants
Single low-intensity stressors across life domains with support	Recovery	Recovery stabilization	*Non-vulnerable*	Aline, Vanina, Paule, Elisa, Leonie, Alizée
Multiple moderate-intensity stressors across life domains with support	Recovery with adjustments	Recovery stabilization	*Resilient*	Natacha, Rachel, Alexandra, Gisela, Leila, Marie-Jo
Multiple high-intensity stressors across life domains with limited support	Fragile recovery	Renewed stressors	*Vulnerable*	Tania, Sophie, Beatrice, Vivianne, Antoinette, Anouk
Multiple high-intensity stressors across life domains with limited support	Non-recovery	Non-recovery	*Chronically vulnerable*	Martine

Source: Own elaboration

In the following subsections, we outline the specific characteristics of each group across the three phases, offering a detailed discussion of their circumstances and challenges. The final subsection provides a synthesis of these findings, presenting a visual representation of the model for a clearer understanding of the phases and trajectories.

### *Phase 1*. Disruption and Resource Activation

Phase 1 represents the initial stage of lone parenthood and is characterized by a general depletion of financial resources and additional stressors across life domains for most mothers. A major feature defining the different trajectory groups at this initial stage is the intensity and multiplicity of stressors experienced across life domains by lone mothers. Preexisting resources, along with the support provided primarily by family and close friends, were crucial in shaping the severity of this period.

#### Single Low-Intensity Stressors with Support

For lone mothers in the non-vulnerable group, the transition to lone motherhood had a limited negative impact on their lives, typically facing low-intensity (single) stressors, and benefited from social support primarily from close relatives and friends. Before this transition, they had accumulated significant resources, which played a protective role in mitigating the potential challenges associated with becoming a lone mother. All these mothers were employed at 80% or more and had (highly) qualified positions with very good work conditions. At the same time, most of them were able to compensate for the direct loss of their partner’s income: either by receiving stable alimony from their ex-partners (Aline and Paule) or by increasing their employment rate (Vanina, Aline).

A good example is Léonie, who held a stable public-sector job that offered workplace childcare. As she explained, one of her first priorities upon becoming pregnant was securing a daycare spot: “Since I work at the Institution in the health sector […] I was given priority over everyone else” (Léonie, Wave 1). This formal support, combined with help from her family, eased her transition to lone motherhood.

These mothers encountered only a few low-level stressors across different life domains: most faced minor schedule-related issues and combined formal care services with an informal childcare network, including family members and often neighbors or acquaintances. Regarding the relationship and the financial and care support of their partners, we encountered a diversity of situations. In Paule and Aline’s case, their ex-partners were highly involved financially and in terms of care since the beginning. Vanina’s partner, however, was irregularly involved, and their relationship was characterized by a permanent negotiation dynamic, through which Vanina tried to shape the father’s involvement with their son. Finally, Elisa and Léonie’s ex-partners were completely absent, and neither alimony nor visit arrangements were set.

A characteristic of this group is the fact that they received support from their families. Given that their financial situations were already good and relatively stable after becoming lone mothers, family support primarily focused on childcare. Friends and acquaintances, including neighbors and babysitters, supplemented this support.

#### Multiple Moderate-Intensity Stressors Across Life Domains with Support

The women with resilient trajectories encountered more intense stressors across multiple life domains. They transitioned to lone motherhood with limited accumulated resources, most of them combining part-time jobs with academic pursuits. Although this arrangement was manageable before the transition, the arrival of a child exposed its limitations. These mothers faced additional moderate stressors related to housing and the relationship with the biological father, often completely absent or lacking caregiving and financial involvement.

A defining feature of the women in this group is the crucial support they received from their families. Childcare responsibilities—often a major source of stress for lone mothers—were manageable for these women owing to significant involvement from their relatives. Marie-Jo, for example, managed to juggle an 80% workload and undergraduate studies by relying heavily on her aunt, who lived nearby. “She really helped me a lot […] when I had exams, or when I went to the library to study, it wasn’t easy. I was really very lucky […] she gave a lot of her time during that period” (Marie-Jo, Wave 1). Now that her child is older, her aunt remains a source of occasional support during holidays. This example illustrates how intensive kin involvement could compensate for limited resources and allow these mothers to sustain demanding work–study arrangements.

Support from personal networks was often combined with formal childcare services (Rachel, Alexandra, Natacha), informal care (e.g., babysitters) (Gisela), or financial assistance from religious associations (Natacha). In the short term, this support eased transitions and prevented greater precarity, while in the long-term, staying engaged in education was key to securing stable, better-paying jobs.

#### Multiple High-Intensity Stressors Across Life Domains with Limited Support

A distinctive feature of the group with vulnerable trajectories is that the women experienced intense, overlapping stressors across multiple life domains—often simultaneously—and with limited support. Many faced unstable employment, precarious housing, and highly conflictual or violent relationships with their ex-partners. These stressors were frequently accompanied by deteriorating mental health**,** including exhaustion, burnout, and symptoms of depression, sometimes extending to crises within the family such as a child’s suicide attempt. And informal support was often absent or inconsistent, leaving them with little room to navigate the demands of lone motherhood. Table 2 summarizes the situation experienced by the women in this group at the beginning of their trajectories across life domains and the support they received.

**Table 2.** Overview of Lone Mothers’ Situation Across Life Domains and Support Received

Participants	Life domains				Support
	Employment status	Housing	Relationship with the child’s biological father	Mental health	
Tania	Unemployed	In a studio with acquaintance, poor conditions; later in a shelter	No paternal recognition, and no paternal involvement	Burnout	Institutional support
Sophie	Permanent employment at 80%	Insecure housing	Violent ex-partner, social services intervention	Trauma; suicide attempt by the child	Emotional support from friends
Vivianne	Permanent employment at 55%	In a cooperative, good housing conditions	Violent ex-partner, social services intervention	Exhaustion	Very limited from neighbors
Antoinette	Unemployed	At ex-partner family apartment (temporary)	Emotional abuse and bullying directed to the children	Exhaustion	Punctual from relatives
Béatrice	Unemployed	At her brother (temporary)	At a distance conflictual relationship	Not specified	Temporary family support
Anouk	Permanent employment at 80%	At parents' house, good housing conditions	Conflictual relationship—Ex- partner alcoholic and putting the child well-being at risk	Depression	Strong financial/practical support from parents
Sylvie	Unemployed	Poor housing conditions (temporary)	At a distance conflictual relationship	Not specified	Financial from her father

Source: Own elaboration

Tania’s case illustrates the accumulation and simultaneity of stressors characteristic of this group. At the beginning of her trajectory, while still pregnant, she was unemployed, living in highly unstable housing, and had no support from the child’s father. She described her precarious situation as follows:

“I was living in a studio with a man who was never my boyfriend… we slept in the same room, and I… I slept on a sponge mattress, a thin mattress like that… and after a while, I could feel the floor underneath me”.

At 7 months pregnant, she contacted a shelter, only to be turned away:

“They said, “But madam, you are not an abused woman, we don’t see this as an emergency.” I don’t understand… We’re in a difficult situation… I was staying at someone’s place… He was kind, he gave me a place to stay… Otherwise, I really didn’t know where to go” (Tania, Wave 1).

After insisting, Tania eventually obtained a place in a shelter and, following the birth of her child, began to receive more consistent institutional support. Her case illustrates how lone mothers in this group transitioned to lone parenthood from a precarious position, facing overlapping stressors and very limited support. More broadly, the convergence of difficulties across multiple life domains meant that women in this group started their lone mother trajectories under particularly fragile conditions, with little room to stabilize their situations at the outset.

#### Multiple High-Intensity Stressors Across Life Domains with Limited Support

Martine exemplifies a case of chronic vulnerability. She was already facing long-term unemployment and mental health issues before becoming a lone mother. Her partner’s high income had previously ensured financial stability, but following their separation, she lost all institutional and legal support^11^.

“Do you not qualify for unemployment benefits as a self-employed worker?

Yes… No, I don’t, but my eligibility period ended a long time ago. I’m living off a small savings fund that is melting away like snow in the sun—it comes from my father’s passing. But it was supposed to be a reserve, you know [...]

So, do you feel like you’re not really receiving any institutional support?

No. But at some point, I might get some. I went to see the Hospice [in reference to the Hospice Général, Geneva’s public welfare institution] last year to get a sense of the situation. They just pick up where unemployment benefits left off, but honestly, I feel like they are completely overwhelmed” (Martine, Wave 1).

After giving birth, Martine faced overwhelming childcare demands without access to affordable alternatives. Financial constraints made babysitters barely affordable, and she struggled to pay rent. Her preexisting mental health issues deteriorated, culminating in postpartum depression and a severe crisis requiring a 6-month hospitalization. Without family or close friends for reliable support, she relied on occasional help from acquaintances—but this inconsistent assistance failed to offset the psychological and material burden of her situation. Martine’s case illustrates the severe consequences of navigating lone motherhood without institutional or social support, placing her in a highly vulnerable position with lasting effects on her well-being.

### *Phase 2*. Steadying the Ship: Navigating the Adjustment Phase

In Phase 2, most lone mothers accumulated new resources and experienced an overall improvement in their situations^12^. However, while this accumulation of new resources was more robust for those in the non-vulnerable and resilient trajectory groups, for lone mothers with vulnerable trajectories, it represented a *fragile recovery*. This distinction, although subtle at this stage, is crucial for understanding the next phase, in which the divergence between the vulnerable trajectories and both the non-vulnerable and resilient trajectories becomes more critical.

#### Recovery

Most lone mothers with non-vulnerable trajectories recovered quickly from the limited stressors they experienced in the previous phase. By this stage, most of these women maintained their mid- to high-skilled jobs, working at 80–100%. However, some made notable adjustments to their employment situations (e.g., reducing working hours and combining employment with other professional projects), reflecting their capacity to prioritize personal and professional development.

Additionally, most of these women began new romantic relationships during this phase, although the stability and duration of these relationships varied. A particularly notable case is Léonie, who repartnered shortly after transitioning to lone motherhood. At the time of her child’s birth, she lived in a small two-room apartment in Lausanne, sharing a room with her son. She later moved in with her new partner: *“*We decided to get an apartment together… now we have a bigger place, and we live as a family of three, four… plus the cat” (Léonie, Wave 2).

Childcare demands for these mothers decreased significantly during this phase: their childcare networks either remained strong or even strengthened over time (e.g., due to repartnering), and their children grew more autonomous and began attending school. In Paule’s case, childcare demands remained significant throughout this phase primarily because she partially lost her childcare network after moving to another town.

For those mothers whose ex-partners were already involved in their children’s daily lives (e.g., Aline and Paule), this involvement continued steadily in this phase. For others, such as Elisa and Léonie, whose ex-partners were absent in the previous phase, this absence persisted. An exception was Vanina, whose ex-partner increased his involvement in childcare, although his financial contributions remained inconsistent.

#### Recovery with Adjustments

Lone mothers in the resilient group saw significant improvements in financial stability, support systems, and reduced childcare pressures. These gains were largely driven by completing their studies and securing stable mid- to high-skilled jobs (Rachel, Natacha, Leila, Alexandra), as well as forming new partnerships and cohabiting, which provided financial and practical support (Gisela, Leila, Marie-Jo). Additionally, the passage of time naturally eased childcare demands. Throughout, family support remained crucial, especially for practical childcare assistance.

However, ongoing stressors and the need for continuous adjustments illustrate the complexity of their trajectories. Repartnering was not always a source of stability: Sarah separated from her new partner, and Marie-Jo went through a second divorce after remarrying and having another child. Persistent conflicts with ex-partners remained a source of stress for some mothers. Leila, for instance, struggled with her child’s adaptation to her new family structure.

Gisela’s experience highlights how the relationship with ex-partners often constitute an important source of stress for lone mothers. She feared her ex-partner might abduct their children, prompting her to pursue legal action regarding custody and financial responsibilities. At one point, her ex-partner threatened to take the children back to his country of origin, where, under local laws, full parental rights would be granted to him. *“*So, it is a real threat,” she said, clarifying that her deeper concern was not direct abduction but the possibility that “he could just hand them over to his family, and they would take care of them—that’s the real risk I see” (Gisela, Wave 2).

Despite living nearby, her ex-partner did not see the children regularly and failed to pay child support for over a year and a half. Legal action had been initiated, but in the meantime, “the payments have completely stopped, and that’s what really concerns me.” Beyond the financial impact, Gisela highlighted the emotional burden of being the sole caregiver:

“The worst part is being the one who has to cover everything in the background. And then their dad comes along: “Oh look, Dad gave me 10 francs! I’m so lucky! He’s so nice!” […] It’s exhausting. It’s frustrating” (Gisela, Wave 3).

This case underscores how even in recovery phases, lone mothers continue managing complex challenges—including unresolved conflicts, inconsistent co-parenting, and legal battles—that demand ongoing adjustment and resilience.

#### Fragile Recovery

Lone mothers with vulnerable trajectories experienced a more fragile recovery. Although most improved their employment situations, often these remained precarious. Tania, Sylvie, and Antoinette had not yet secured stable employment; Vivianne, despite wanting to increase her hours, remained limited to a 55% contract; Béatrice obtained a 70% permanent job; while Sophie and Anouk maintained their permanent positions. Housing also improved for some, though often in fragile or temporary ways: Tania and Béatrice accessed social housing, and Vivianne benefited from a cooperative apartment. In other cases, external events were decisive—Sophie, for example, secured stable housing after inheriting an apartment, while Sylvie’s inheritance allowed her to move to a better space, temporarily easing her conditions.

However, many of these gains proved short-lived. In cases like Sylvie’s and Vivianne’s, improvements depended on inheritance or time-limited support. Meanwhile, emotional stressors persisted, particularly in relation to children’s fathers, and these often undermined women’s efforts to stabilize their lives. Anouk, for instance, experienced burnout and depression linked to a failed reconciliation attempt with her child’s father, who struggled with alcoholism. Vivianne became entangled in a legal procedure related to the father’s violent behavior. Tania continued to face the challenge of involving a father who still refused to recognize his child. Sylvie tried to sustain a distant father–child relationship, which added further strain. These burdens accumulated on top of the initial difficulties of lone parenthood and affected not only the mothers’ well-being but also their children’s situations.

A particularly complex example is Béatrice’s case, which shows both the opportunities and fragilities of this trajectory. During this phase, she made significant progress: securing a stable job, relocating to the German-speaking part of Switzerland, finding better childcare, and beginning to explore new relationships. Yet soon after, her son’s situation deteriorated sharply. He had already experienced behavioral difficulties at school, limited friendships, and an ambiguous relationship with his father, who remained in Africa after the separation. Although Béatrice consistently tried to ensure father–child relationship, something that was very important for her, her son resisted, and she often felt torn between encouraging this relationship and respecting his wishes. Against this background, she described this period as “a descent into hell,” as his behavioral crises escalated:

“There was an extremely serious incident [a behavioral problem at school]. It happened maybe three times… and some people had already been alerted. They were the ones who told me: He needs to be somewhere with professionals who can monitor him because this is a situation of insecurity” (Béatrice, Wave 2).

Her son was subsequently hospitalized in a mental health center, where he remained under psychiatric supervision for several weeks. According to Béatrice, these struggles persisted over time and were primarily linked to unresolved trauma from his uprooting from Africa and the absence of his father, combined with what she suspected were high intellectual abilities. As she explained:

“We think it’s due to a combination of factors. One is the uprooting from Africa… it’s a wound that has never healed. And second, I never really wanted to go through the courts for pseudo-diagnosis… but we think he might be gifted” (Béatrice, Wave 2).

Béatrice’s case highlights how even substantial material progress can be destabilized by a difficult relationship with her ex-partner and by her son’s emotional and behavioral difficulties. Her experience illustrates the precarious balance of fragile recovery, where improvements in employment or housing are continually undermined by ongoing challenges across life domains. More broadly, the fragile recovery trajectory shows that lone mothers’ situations cannot be fully understood without considering the interplay between material conditions, complex relationships with ex-partners, and children’s outcomes—all of which may generate stress and tensions with serious consequences for mothers and children’s mental health.

#### No Recovery

Martine’s case stands out as an exception. Unlike most others, she experienced little to no recovery and instead faced a severe mental health crisis while relying heavily on social assistance. She attributed her 2017 breakdown—several years after becoming a lone mother—primarily to her employment situation. Enrolled in a job reintegration program, she had secured a 50% position at a museum, which she viewed as a crucial step toward regaining material stability. However, after a long and uncertain application process, she was not selected for a permanent position. The disappointment triggered a major depressive episode, leading to a 6-week hospitalization:

“It was a really difficult situation… I waited for about ten months, and they were still slow to respond. I did everything I could to keep that job. But in the end, I wasn’t selected. And then, I fell into a deep depression. Just that alone. I was hospitalized for six weeks” (Martine, Wave 3).

This breakdown deepened her ongoing sense of vulnerability, especially as her daughter entered adolescence and emotional and financial pressures continued to mount. Martine explained how she felt increasingly disconnected from her peers, overwhelmed by the complexity of her situation, and unable to fulfill the role she believed was expected of her at this stage of life:

“Sometimes things go well, sometimes less so… I feel pretty overwhelmed. It’s complicated because I’m far removed from that stage of life. The world has changed” (Martine, Wave 3).

Later in the interview, she clarified that by “stage of life” she meant being able to act as both the financial and care provider for her daughter. Instead, she found herself unemployed, emotionally exhausted, and unable to be fully present for her child as she entered adolescence. Her words reflect both the isolation of living with chronic vulnerability and the perception that time was moving forward for others while she remained stuck in crisis management. During her hospitalization, her biological child stayed with her ex-partner—whom she identified as her only strong source of support during this period. Overall, Martine’s case illustrates the absence of recovery, showing how unresolved employment insecurity, accumulated pressures, and fragile support networks can converge into long-term vulnerability.

### *Phase 3.* Settling Unevenly: Late Adjustment and Unequal Recovery

Phase 3 represents the most advanced stage of lone mothers’ trajectories, occurring on average around 10 years after their transition, and is defined by two distinct major dynamics. For those with non-vulnerable and resilient trajectories, this stage marked the long-term stabilization of the recovery initiated in the previous phase. By contrast, lone mothers with vulnerable trajectories faced a renewed period of pronounced vulnerability, often accompanied by severe mental health challenges. Meanwhile, Martine, the lone mother with a chronically vulnerable trajectory, cognitively adapted to her prolonged extreme precarity, although she did not experience recovery.

#### Recovery Stabilization

The key dynamic in this phase is recovery stabilization for lone mothers in both non-vulnerable and resilient trajectories. Although those in the non-vulnerable group experienced a less dramatic recovery due to stronger starting positions, both groups maintained long-term equilibrium through resource accumulation, life adjustments, and sustained improvement.

Léonie exemplifies long-term stability among non-vulnerable lone mothers. With strong initial resources and a stable situation, her main stressor had been the absence of her child’s biological father. Over time, as her relationship with her new partner strengthened, this concern diminished. Eight years after her transition, she and her partner began legal proceedings to adopt her son, highlighting both material security and long-term emotional and family cohesion. “Paul has been there since Nicolas was 8 months old, so for him, he’s only ever known him as his father,” she explained. “Now we’re starting the process for Paul to adopt him.” (Léonie, Wave 5).

Similarly, Rachel’s story illustrates long-term stabilization among resilient lone mothers. With strong family support and a fulfilling job in the information sector, her situation had significantly improved by Phase 3. Her daughter, now 18, had become largely autonomous, allowing Rachel to reduce her caregiving duties and focus more on work and personal well-being. She maintained a satisfying “living apart together” relationship and a cooperative co-parenting arrangement: “The father lives nearby, really close… everything’s going really well—everyone’s really happy.” (Rachel, Wave 5).

#### Renewed Stressors

Vulnerable lone mothers faced renewed stressors across multiple life domains, marking a setback in their fragile recovery. These crises, occurring 10 to 14 years post-transition, stemmed from employment struggles (Béatrice, Tania, Sophie, Vivianne), unresolved conflicts with ex-partners (Anouk), or, in some cases, no clear trigger (Sylvie). For some, the COVID-19 crisis compounded these challenges by disrupting employment and intensifying uncertainty, while also adding further strains such as increased mental burden from frequent decision-making, reduced support due to distancing norms, and fewer opportunities for sociability. While these challenges were shared by many parents during the pandemic, for lone mothers they acted as a multiplier of the usual strains associated with raising children without a partner, thereby exacerbating preexisting inequalities (Sánchez-Mira et al., 2021, 2022).

Mental health impacts varied, from extreme exhaustion (Sylvie) to burnout requiring sick leave (Béatrice, Anouk, Sophie) or prolonged absence from work (Vivianne). Some faced multiple crises in quick succession. For example, Sophie accepted a full-time job in 2018 after achieving stability but soon suffered burnout, leading to extended sick leave. Upon returning in 2021, workplace bullying triggered another episode.

“Yes, it was a burnout, so it was quite intense, actually. I would say, in relation to your research, that I felt extremely vulnerable, both in terms of my physical and mental health. And it happened at a time when I needed to be a strong support for my children. Honestly, these past few years—with the lockdowns and Elyas starting university studies entirely remotely—have been really tough. He had a very difficult year. And their father, who was already not very present, was even more absent because of COVID—he was stuck in a City in a Country of Europe 4 and never came. So, it was really at the moment when my boys were transitioning into adulthood that they had to…” (Sophie, Wave 5).

Although Sophie left this sentence unfinished, the context of her narrative suggests that she was referring to her sons’ need to navigate the transition to adulthood largely autonomously, and under the strain of additional family-related difficulties and limited maternal support. This illustrates not only the challenges faced by children but also the mental burden carried by lone mothers, who are acutely aware of how their situation may negatively affect their children’s trajectories.

Béatrice experienced burnout and underwent psychiatric treatment in 2021, citing work overload and her son’s school difficulties. Tania, although not diagnosed with burnout, struggled to balance work and childcare during the COVID-19 pandemic, reducing her hours to 50% by 2021. Sylvie, while not on sick leave, reported extreme exhaustion, worsened by financial strain and her son’s newly diagnosed learning difficulties. Unlike more resilient lone mothers, none of the mothers in this group cohabited with a partner, lacking daily support. Additionally, unresolved conflicts with their children’s fathers added moral dilemmas, further intensifying their mental and emotional strain.

These renewed stressors illustrate how recovery among vulnerable lone mothers remains fragile and subject to reversal. External shocks—whether global (e.g., COVID-19) or personal (e.g., job loss or parenting strain)—can rapidly undermine hard-won stability. For many, these late-stage crises reignited earlier vulnerabilities, revealing the ongoing need for sustained structural, emotional, and institutional support long after the initial transition to lone motherhood.

#### No Recovery

By this stage, Martine had fully resigned herself to chronic vulnerability, having abandoned the search for stable employment after years of repeated rejection. The emotional toll of continuous setbacks, coupled with employer biases against older workers and her non-linear career path—marked by periods abroad, shifts in professional trajectory, part-time work combined with care duties, and subsequent long-term unemployment after returning to Switzerland—led her to conclude that reintegration into the labor market was no longer a viable option. Instead, she turned to irregular artistic projects—such as editing concert videos or supporting music schools—which brought personal satisfaction but lacked financial stability.

Reflecting on her decision, Martine explained: “Honestly, I completely stopped looking for work. The last time I tried—despite the job being practically made for me—I just couldn’t handle being rejected over and over again.” She occasionally earns money from creative work and no longer attempts to find formal employment. “I do what I enjoy, even if there’s no money… sometimes there is, sometimes there isn’t. But at my age, I’ve given up on the idea of a traditional job.” She described how repeated failures led to a loss of self-confidence, especially in a system that she sees as rigid and punishing to anyone who does not “fit the mold.”

“I lost so much confidence after constantly looking and never finding… I experienced that for a long time, and it played a huge role in my depression.” (Martine, Wave 5). Now approaching 58, she survives on social assistance and focuses on what brings her meaning: *“*I’ve decided to stop searching and just live. I do the things I enjoy. That actually helped me rebuild some confidence.”

Martine’s case underscores how chronic vulnerability and prolonged exclusion from the labor market can lead to resignation rather than recovery—especially when the system offers few meaningful reintegration pathways for older women with unconventional profiles.

### Synthesis of the Findings

**Figure 1.**
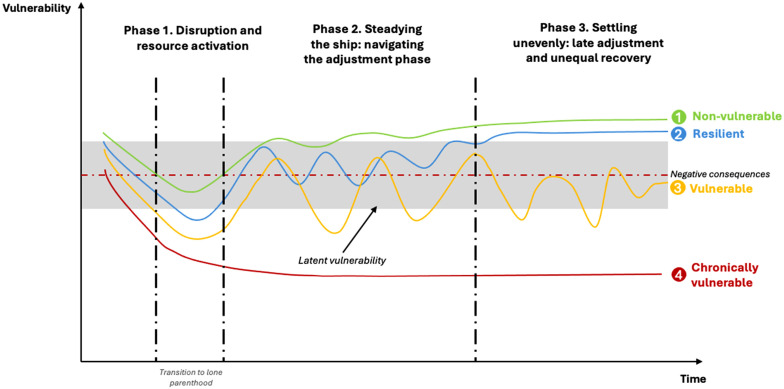
Model of Lone Parenthood Phases and Trajectories. Source: Own Elaboration Inspired by Orsholits (2020)

Based on the previous sections, Figure 1 presents a synthesis of the findings, offering a general dynamic and empirically grounded model of the different phases and trajectories of lone parenthood. The *x*-axis of the graph represents time, illustrating the progression of lone parenthood experiences over more than 10 years. Although the women in our sample transitioned to lone motherhood at different points in their lives, this model suggests that they all experienced some form of these three ideal-type phases.

The *y*-axis represents vulnerability outcomes, indicating the well-being or stability of lone mothers. Higher values on this axis suggest better outcomes, such as stability and resilience, and lower values indicate negative consequences, such as persistent difficulties and vulnerability.

The transition to lone parenthood is represented by two dashed lines, indicating a period of time rather than a single moment, suggesting that the transition to lone parenthood is not an abrupt event but rather a gradual process. The spacing between these lines highlights that the challenges and adjustments associated with this transition may differ in duration and intensity across individuals.

The red dashed line marks the boundary between negative consequences and more stable outcomes, highlighting the contrast between those who manage to recover and those who continue to experience stressors across life domains. A key feature of the graph is the gray shaded area, which represents a threshold of *latent vulnerability*. This area signifies a critical zone in which lone mothers may experience instability, with potential risks of deterioration over time. If a trajectory remains in or below this zone, it suggests prolonged hardship and difficulty in overcoming challenges.

## Discussion

By following lone mothers over time and proposing a model of lone parenthood, this study contributes to the literature in five key ways. First, the findings challenge the assumption dominant in cross-sectional research that lone parenthood is uniformly associated with poverty and negative outcomes. When examined through a biographic and family development lens, lone parenthood appears to be more diverse, nuanced, and continuously shaped by the ongoing interaction between stressors and resources across life domains that may or may not produce temporary or chronic vulnerabilities. However, in line with previous research (Hübgen, 2018; McLanahan, 2004; McLanahan & Jacobsen, 2014), lone mothers’ situation before the transition seems to be crucial: those with stronger initial resources tend to experience fewer stressors and recover more quickly, whereas those with fewer resources face greater challenges and prolonged vulnerability.

Second, although our findings recognize the importance of financial stressors in lone parenthood (Brady & Burroway, 2012; Broussard et al., 2012), they extend further by highlighting the more diverse and evolving nature of the challenges faced by lone mothers. Challenges can arise in different life domains (e.g., the relationship with the biological father, childcare demands, or health-related issues); some naturally decrease in intensity over time (e.g., childcare), and others may persist or even intensify (e.g., mental health issues). Even for those who improve their overall situation, unresolved long-term stressors in specific life domains—such as relationships with biological fathers—can offset the positive general gains in other life domains (e.g., Gisela and Marie-Jo) (Moles et al., 2024). Ongoing struggles can create a state of continuous decision-making, in which lone mothers must constantly manage and reallocate limited resources, leading to persistent mental burden and psychological strain (Dinescu et al., 2018; Kühn, 2018; Sánchez-Mira et al., 2022; Struffolino et al., 2016).

Third, a key characteristic of different lone mother trajectories is the interplay between the intensity and simultaneity of stressors across life domains. In our sample, chronically and vulnerable lone mothers experienced high-intensity stressors that occurred simultaneously across multiple domains, often creating a “negative loop” with potential long-term consequences for their well-being that was difficult to break. This finding aligns with recent research highlighting that a concentration of critical life events and turning points in time can have a negative long-term impact on individual and family well-being (Comolli et al., 2024).

Fourth, this study highlights lone mothers’ agency in managing adversity, showing that most gradually improve their overall situation—an aspect largely overlooked in previous research. A key element of this recovery is the means and timing of resource mobilization. In the early stages, lone mothers primarily rely on personal networks (close relatives and friends), as these provide the quickest and most accessible forms of support to meet urgent needs (e.g., childcare). Institutional resources are usually considered at a later stage, once immediate challenges are stabilized, and are mobilized as an additional rather than substitutive source of support. Although this sequence is not always distinct, as the stages may overlap, it supports previous research emphasizing the importance of personal networks in immediate assistance (Harknett, 2006, 2011; Keim, 2018; Lin, 2002). Access to institutional resources, however, often entails greater costs in terms of time, availability, and cognitive effort, especially for non-normative families. While social services and family support for lone parents are (limited) available, it may take time to identify which policies and benefits they are eligible for. Moreover, actually obtaining support requires considerable effort and energy, as it involves complex bureaucratic procedures particularly in Switzerland, where services are organized at the cantonal level and regulations vary from one canton to another—further complicating the process and, paradoxically, turning the very act of obtaining welfare benefits into an additional stressor that accumulates on top of the difficulties which led mothers to seek institutional support in the first place (Larenza, 2019; Nieuwenhuis & Maldonado, 2018; Seefeldt & Sandstrom, 2015; Stack & Meredith, 2018).

Finally, this study challenges the notion of a linear progression in family recovery following a crisis such as separation or divorce (Hill, 1958; Van Gasse & Mortelmans, 2020). By introducing the concept of latent vulnerability as a process of *fragilization* (Spini et al., 2013, 2017), we show that recovery can be precarious and unstable. In the case of vulnerable mothers, this instability may lead to renewed, more manifest forms of vulnerability in later stages. Importantly, rather than resulting from a sudden “last straw” effect, the stressors experienced by vulnerable lone mothers in Phase 3 are better understood as the outcome of an accumulation of disadvantages over time (Dannefer, 2003, 2020). The already limited availability of resources at the beginning of their trajectories, together with ongoing pressure to adapt to changing circumstances—combined with financial and emotional strain—contributed to heightened levels of anxiety, depression, and burnout, particularly for those with more fragile trajectories. These challenges often remained invisible; nevertheless, they can be critical in shaping mothers’ capacity to cope and recover over time.

## Limitations and Conclusions

This study presents several limitations, which suggest valuable directions for future research. First, despite efforts to ensure diversity during participant recruitment, individuals from lower socioeconomic backgrounds remained underrepresented in the sample. Nonetheless, the inclusion of participants from mid-to-high socioeconomic status offered the opportunity to examine how vulnerability can emerge not only from material deprivation but also from challenges related across different levels of the life course—such as relational or institutional contexts.

Second, although this study underscored the role of close relatives and friends in providing support—especially during the early stages of lone mothers’ trajectories—further research could deepen this understanding by examining the structure and composition of lone mothers’ social networks in more detail. This line of inquiry could be enriched through qualitative approaches that explore how lone mothers perceive the support they receive and the meanings they attribute to various forms of assistance.

Finally, although the present study explored the subjective experiences of lone mothers across diverse groups and trajectory stages, future qualitative research could illuminate the emotional and affective dimensions of these experiences—for example, feelings of relief, freedom, or empowerment associated with the transition to lone parenthood. Such work would contribute to a more nuanced and dynamic understanding of how lone parents navigate and interpret their evolving circumstances, thereby enhancing the complexity and richness of representations of lone parenthood.

Overall, this study has shown the importance of examining lone parenthood through a life-course and vulnerability perspective. By analyzing lone parenthood over time and accounting for the dynamic interplay between stressors and resources across life domains, this analysis reveals that lone parents’ experiences are far more heterogeneous and nuanced than those suggested by most previous research.

Furthermore, the findings hold important implications for social interventions, offering insights for policymakers. In particular, they underscore the need for policies that acknowledge the multifaceted nature of the challenges lone parents may encounter. These challenges extend beyond financial constraints to include relational dimensions—such as ongoing interactions with the nonresident parent—which often intersect with material disadvantage and contribute to greater strains.

First, interventions during the initial transition to lone parenthood (Phase 1) are crucial, as they can help prevent the accumulation of disadvantage over time. Such measures should not only address material dimensions but also the relational strains that many lone-parent families face (Moles-Kalt et al., 2024). Experiences from other contexts illustrate the value of proactive support at this stage. For example, in Norway, divorcing parents with children under 16 are required to attend free public mediation at family counseling offices, with the aim of centering the child’s best interests and facilitating parental communication in custodial agreements (Eikrem & Jevne, 2022). Similar interventions adapted to the Swiss context could help lone parents manage relational as well as economic pressures at the outset of their trajectories.

Second, attention must be paid to the design of social policies themselves. This is particularly relevant in Switzerland, where the availability of services and eligibility criteria differ significantly across cantons. Such fragmentation not only complicates mothers’ navigation of the welfare system but can also create inequalities of access and, paradoxically, reinforce vulnerability instead of reducing it.

Finally, our results point to the importance of combining *universal public policies* with *targeted interventions* that accompany the most vulnerable families over time. Previous research has emphasized the importance of universal measures such as affordable and flexible childcare services or parental leave (Larenza, 2019; Nieuwenhuis & Maldonado, 2018). Yet these should be complemented with more intensive and sustained forms of support targeted at those experiencing persistent instability across life domains, for whom universal measures alone are insufficient.

## Supplemental Material

Supplemental Material - Rethinking Maternal Gatekeeping Navigating Lone Parenthood Over Time: A Qualitative and Vulnerability Life-Course Approach Supplemental Material for Rethinking Maternal Gatekeeping Navigating Lone Parenthood Over Time: A Qualitative and Vulnerability Life-Course Approach by Benjamin Moles and Laura Bernardi in Journal of Family Issues.

## Data Availability

Longitudinal qualitative data from the first four waves of the project, the *Multiple Paths of Lone Parenthood (2012–2022)*, are available online in SWISSUbase (Ref. 13853).*

## Notes

1. For a review of the applications of similar stress models at the individual, family, and community levels, see Bonanno et al. (2015).

2. In 2018, shared custody arrangements—defined as a 30%–70% or more equal division of time—were estimated at only 14.1% (Recksiedler and Bernardi, 2023). To address this imbalance, a 2017 reform was introduced, requiring judges to consider shared custody regardless of parental willingness.

3. Maintenance payment supports are calculated based on the average minimum expected cost and not on the real related expenses; thus, typically, the alimony payments do not suffice (Larenza, 2019). Furthermore, in Switzerland, at least 20% of the noncustodial fathers violate alimony obligations (Struffolino et al., 2020).

4 Data from W1 to W4 have already been published through SWISSUbase and can be accessed upon request.

5. Details on the sample characteristics for W1 and W5 can be found in Appendix A.

6. This characteristic needs to be considered in interpreting the findings, since the variation in material and social resources associated with different educational levels may have influenced their trajectories.

7. Summary of these conceptual and analytical steps is provided in Appendix B. For clarity, the figure presents them as numbered sequential steps; however, the analytical process was *iterative*, involving constant movement between steps to enable a dynamic and reflexive analytical process.

8. We defined stressors as events or conditions that disrupted a lone mother’s stability and coping capacity. Appendix E tables indicate the presence (black bullet) or absence (white bullet) of stressors and resources across life domains. The “partnership” domain was excluded, as all participants were unpartnered during the examined initial phase. A dichotomous approach (white or black bullet) was used for clarity, with nuances discussed in the findings section.

9. While ideal types are constructed on the basis of several cases, we retained the chronically vulnerable trajectory as a distinct type despite it being represented by a single case. Our aim is not statistical representativeness but the analytical generalizability of processes and dynamics. In this sense, ideal types are used heuristically to capture trajectories that may be less common but nonetheless reflect existing social realities. This trajectory illustrates that recovery after the transition to lone parenthood is not universal and that some mothers face persistent vulnerability over time. Retaining this trajectory is important not only for theoretical refinement but especially for policy, as it reflects lone mothers in more complicated situations who may require greater and sustained institutional support over time.

10. The following subsections provide empirical examples that clarify how low-, moderate-, and high-intensity stressors are understood in this study.

11. As a same-sex couple in Switzerland, neither had legal rights over each other’s biological children, complicating their informal co-parenting arrangement.

12. After initially relying on their personal networks during Phase 1, most lone mothers across the four trajectory groups began to access and activate social benefits. These benefits—including housing assistance, participation in professional reintegration programs, family allowances, BRAPA/SCARPA, or children’s health insurance subsidies—functioned as immediate resources to stabilize their situations rather than as long-term reserves for future emergencies. Although *access* to many of these social benefits was linked to the condition of lone motherhood, their effective *activation* often depended on the mothers’ skills, competencies, and social networks. Larenza (2019) explored this issue in depth using participants from the same sample as this study; thus, we do not elaborate further on this dimension.
