# Supplementary material for: Navigating Lone Parenthood Over Time: A Qualitative and Vulnerability Life-Course Approach
Source: J Fam Issues. 2025 Nov 3;47(5):691–727. doi: 10.1177/0192513X251393152 (PMC12991351; doi:10.1177/0192513X251393152)
Supplement: Supplemental Material - Rethinking Maternal Gatekeeping Navigating Lone Parenthood Over Time: A Qualitative and Vulnerability Life-Course Approach [file sj-pdf-1-jfi-10.1177_0192513X251393152.pdf]

APPENDIX A – SAMPLE CHARACTERISTICS AT W1 AND W5

Table 1. ***Sample Characteristics at Wave 1 (2012–2013)***

**Pseud.**	**Year of separation**	**Age of children (years)**	**Age of mothers**	**Occupation (last job if not in employment*)**	**Relationship status**	***De facto* physical custody**	**Trajectory ideal type**
Léonie	2012	Pregnant	35	Social worker	No partner	Sole custody	Non-vulnerable
Elisa	2009	2	40	Educator	No partner	Sole custody	Non-vulnerable
Paule	2009	5	40	Executive assistant	No partner	Sole custody with visitation arrangements	Non-vulnerable
Aline	2011	5 and 8	42	Psychologist	No partner	Sole custody with visitation arrangements	Non-vulnerable
Vanina	2007	8	38	Educator	No partner	Sole custody	Non-vulnerable
Alexandra	2009	2	45	Teacher	No partner	Sole custody	Resilient
Gisela	2010	3 and 5	29	Administrative officer*	No partner	Sole custody with informal and irregular visitation arrangements	Resilient
Natacha	2008/2009	4	30	Psychologist	No partner	Sole custody with informal visitation arrangements	Resilient
Marie-Jo	2008	4	37	NGO sector professional	No partner	Sole custody	Resilient
Leila	2009	5	28	Administrative officer	No partner	Sole custody	Resilient
Rachel	2008/2009	8	33	IC sector professional	No partner	Sole custody with visitation arrangements	Resilient
Sarah	2007	8	32	Student (no information about her last job)	Repartnered, not cohabiting	Sole custody	Resilient
Tania	2011	1	29	Waitress*	No partner	Sole custody	Vulnerable
Sylvie	2009	2	37	Kindergarten teacher*	No partner	Sole custody	Vulnerable
Anouk	2010	3	41	Social worker	No partner	Sole custody with informal visitation arrangements	Vulnerable
Béatrice	2010/2011	6	41	Project manager	No partner	Sole custody	Vulnerable
Viviane	2005/2006	6	49	Teacher	No partner	Sole custody	Vulnerable

Pseud.	Year of separation	Age of children (years)	Age of mothers	Occupation (last job if not in employment*)	Relationship status	*De facto* physical custody	Trajectory ideal type
Sophie	2006	8 and 10	41	Sociocultural animator	Repartnered, not cohabitating	Sole custody with visitation arrangements	Vulnerable
Antoinette	2006	12 and 15	41	Social worker	No partner	Sole custody with visitation arrangements	Vulnerable
Martine	2010	9 and new born	49	Cultural manager (independent)*	Repartnered, not cohabitating	No custody for the first, sole custody for the second	Chronically vulnerable

Source: Own elaboration

Table 2. ***Sample Characteristics at Wave 5 (2022)***

Pseud.	Age of children (years)	Age of mothers	Occupation (last job if not in employment*)	Relationship status	*De facto* physical custody	Trajectory ideal types
Léonie	10	45	Director of service in health sector	Repartnered, cohabiting	Sole custody	Non-vulnerable
Elisa	12	49	Educator	No partner	Sole custody	Non-vulnerable
Paule	15	49	Executive assistant	Repartnered, not cohabitating	Sole custody with visitation arrangements	Non-vulnerable
Aline	15 and 17	53	Psychotherapist	Repartnered, not cohabitating	Sole custody with visitation arrangements	Non-vulnerable
Vanina	18	48	Educator	No partner	Sole custody with visitation arrangements	Non-vulnerable
Alexandra	12	55	Teacher	No partner	Sole custody with visitation arrangements	Resilient
Gisela	13 and 15	40	Consultant	Repartnered, cohabiting	Sole custody with visitation arrangements	Resilient
Natacha	14	40	Psychologist	Repartnered, not cohabitating	Shared custody	Resilient
Marie-Jo	2 and 14	46	Executive Assistant in the education	Remarried, had a second child, and	Sole custody of older child and sole custody of younger child with	Resilient

Pseud.	Age of children (years)	Age of mothers	Occupation (last job if not in employment*)	Relationship status	*De facto* physical custody	Trajectory ideal types
			sector	separated again	visitation arrangements	
Leila	4 and 15	38	Public service professional	Repartnered, cohabiting, child with new partner	Sole custody for the other parent with visitation arrangements for the older child	Resilient
Rachel	18	43	IC sector professional	Repartnered, not cohabitating	Sole custody with visitation arrangements	Resilient
Sarah	18	42	Education sector professional	Repartnered, cohabiting	Sole custody	Resilient
Tania	11	39	Administrative Manager in public administration	Repartnered, not cohabitating	Sole custody with visitation arrangements	Vulnerable
Sylvie	12	46	Extra-curricular school service employee	No partner	Sole custody	Vulnerable
Anouk	13	51	Social worker	No partner	Sole custody with visitation arrangements	Vulnerable
Béatrice	16	50	Director of environmental sector organization	No partner	Sole custody	Vulnerable
Viviane	16		Teacher	No partner	Sole custody	Vulnerable
Sophie	18 and 20	43	Dean of education program in the social sector	Repartnered, not cohabitating	Sole custody with visitation arrangements	Vulnerable
Antoinette	22 and 24	51	Social worker	No partner	Sole custody	Vulnerable
Martine	17 and 8	59	Cultural manager*	No partner	No custody for the first, sole custody for the second	Chronically vulnerable

Source: Own elaboration

	Within cases	Across Cases
**Cross-sectional**	**1) Case description**<br>• *Pen portraits (with "see also links")*<br>• Provisional codes and categories	**2) Case comparison**<br>• Establishing criteria for comparison<br>• Refining codes and categories
**Longitudinal**	**3) Case description**<br>• *Grid analysis*<br>**4) Case process analysis**<br>• Process tracking<br>• *Case history*	**5) Process comparison**<br>• Articulating cases, themes and processes<br>• Types of change and underlaying dynamics<br>• Process tracking<br>• *Case history*<br>• *Life domains graphical representation*

APPENDIX B – CONCEPTUAL ANALYTICAL STEPS TO IDENTIFY STABILITY AND CHANCE OVER TIME

Source: Own elaboration based by Vogl et al., 2017; Thomson, 2007; Lewis, 2007; Neale, 2021, Hollstein, 2021.

APPENDIX C – CASE HISTORY FILE EXAMPLE

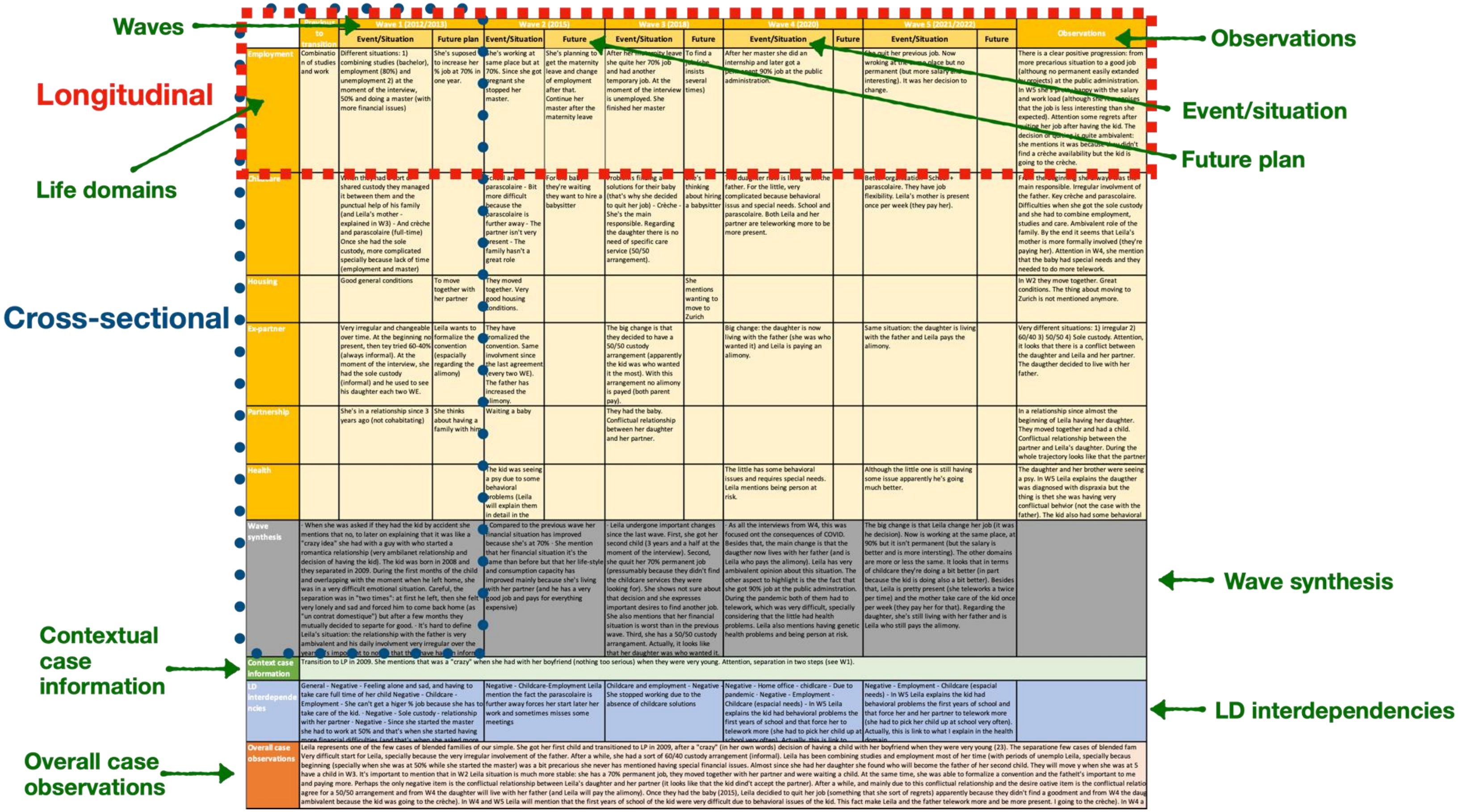

Source: Own elaboration inspired by Vogl et al., 2017; Thomson, 2007; Lewis, 2007; Neale, 2021, Hollstein, 2021.

APPENDIX D – GRAPHICAL REPRESENTATION

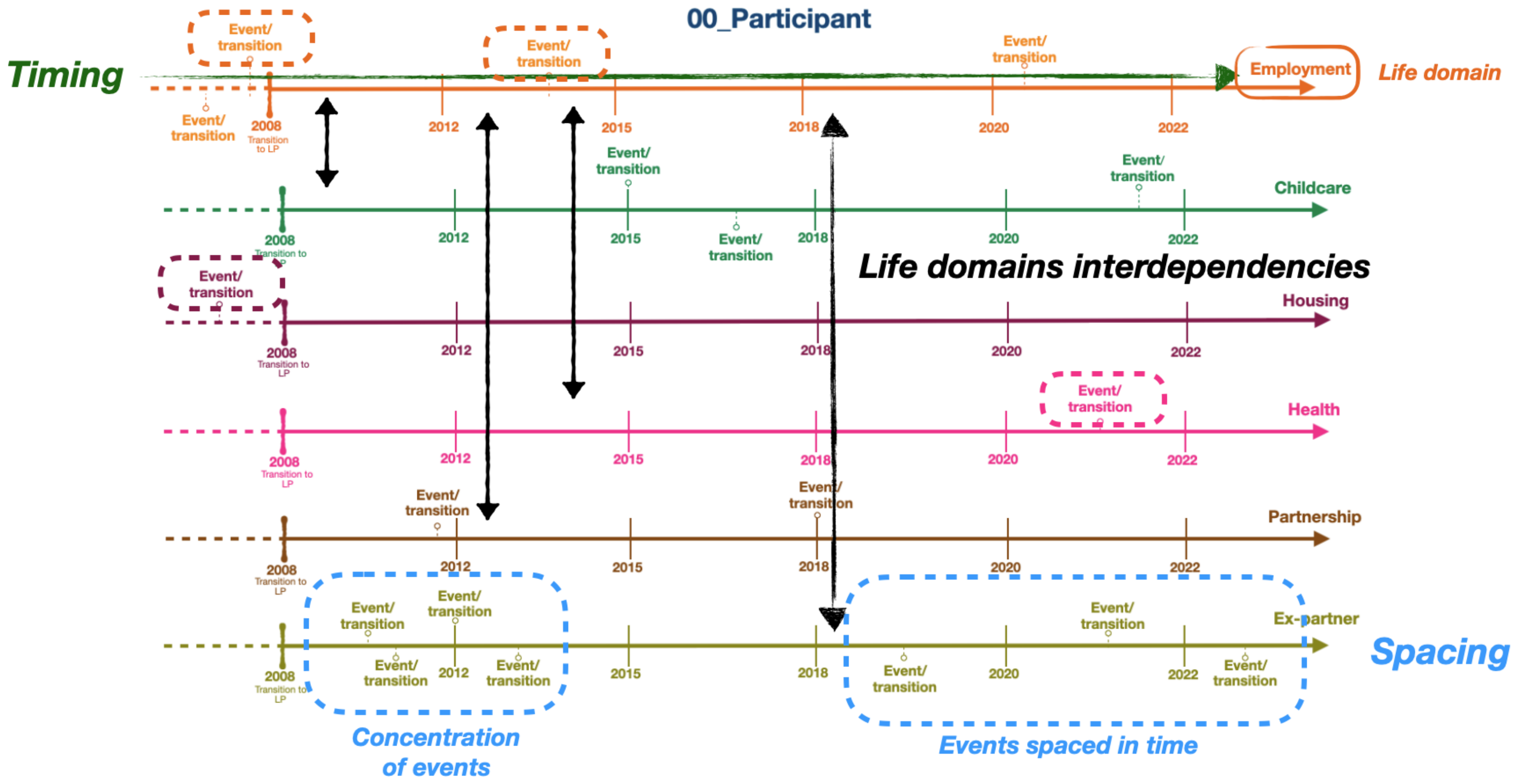

Source: Own elaboration.

APPENDIX E – "CRISP-SETS" INSPIRATION TABLE EXAMPLE

Multiple high-intensity stressors across life domains with limited support

Cases	Stressors across life domains					Support activated		Social assistance
	Employment	Childcare	Housing	Health	Ex-partner	Strong ties (family, close friends)	Weak ties (neighbors, colleagues)	
Sylvie	●	●	●	○	●	●	○	✗
Antoinette	●	●	●	●	●	●	●	✗
Beatrice	●	●	●	○	●	●	●	✗
Vivianne	●	●	○	●	●	○	●	✗
Tania	●	●	●	●	●	●	●	✓
Anouk	○	○	●	●	●	●	○	✗
Sophie	○	●	○	●	●	○	●	✓

● = Presence of a stressor or activated support
○ = Absence of a stressor or activated support

✓ = In social assistance
✗ = Not in social assistance

Source: Own elaboration inspired by Ragin, 1987; Mello, 2014, 2021; Hollstein & Wagemann, 2014.
